# Supplementary figures and images for: Risk factors for contracting malaria in six wards of Mudzi District, Zimbabwe: A case control-study
Source: PLoS One. 2025 Aug 7;20(8):e0329093. doi: 10.1371/journal.pone.0329093 (PMC12331041; doi:10.1371/journal.pone.0329093)

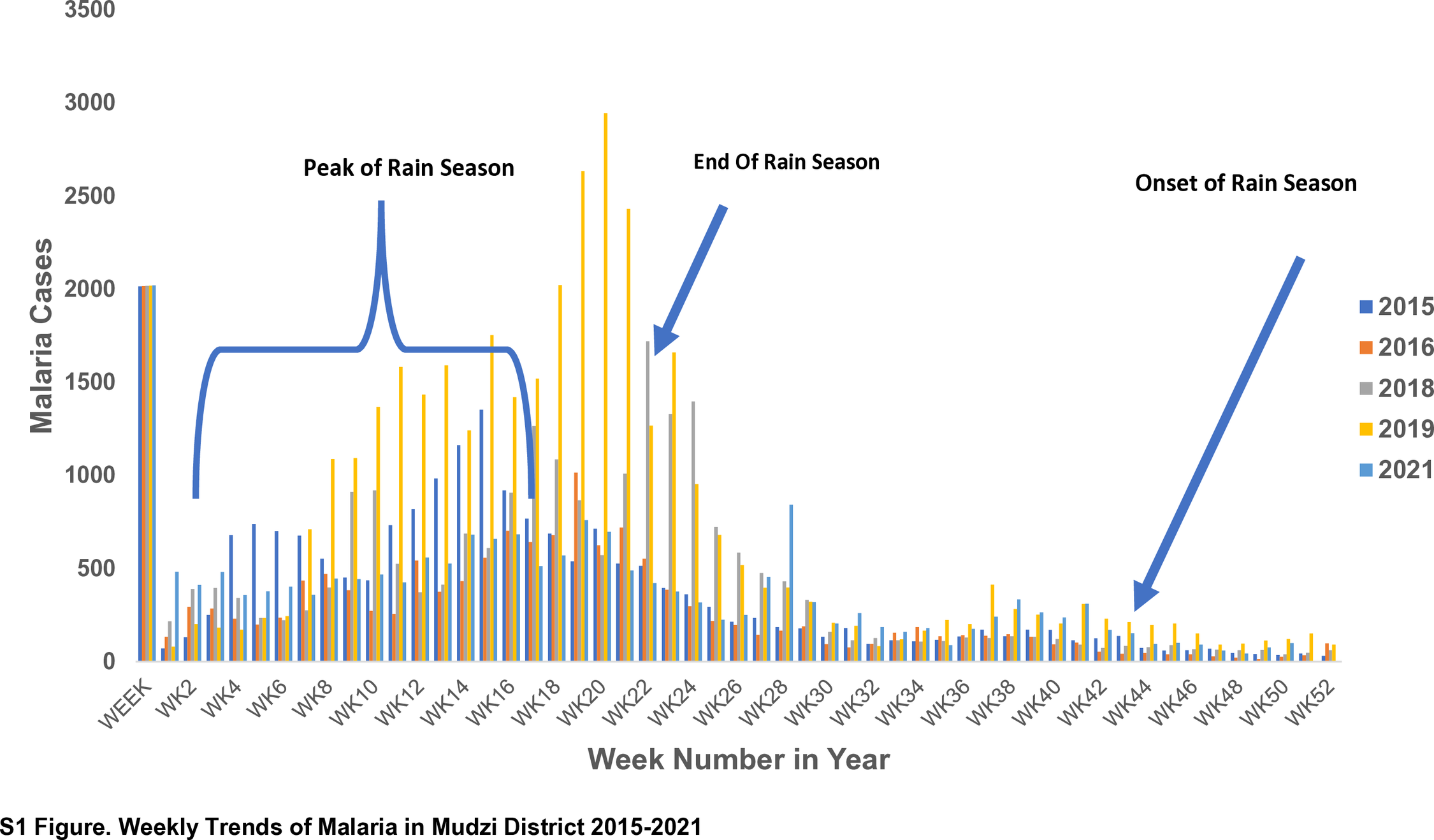

Supplement: S1 Fig — (TIF) [file pone.0329093.s001.tif]

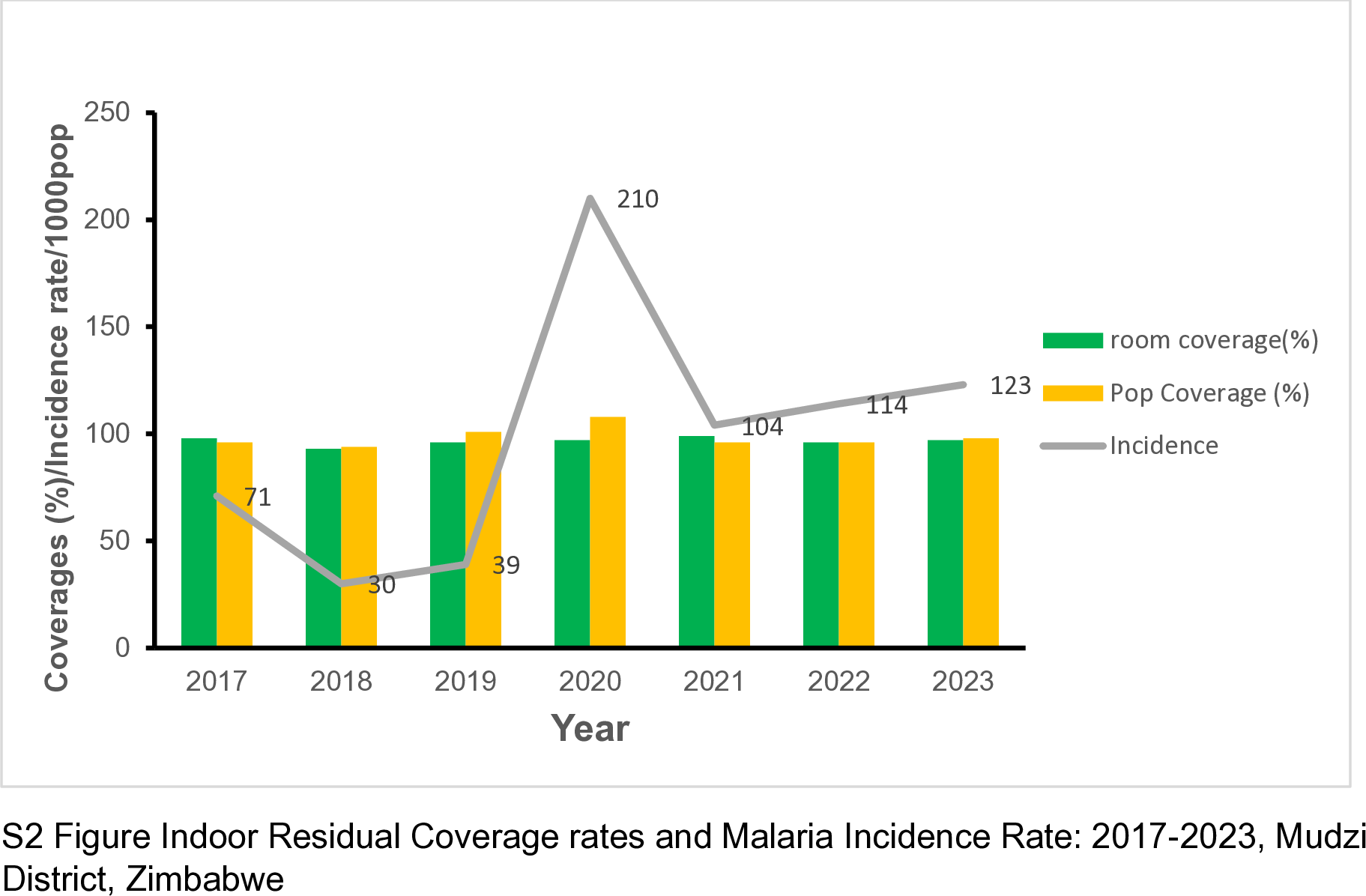

Supplement: S2 Fig — (TIF) [file pone.0329093.s002.tif]

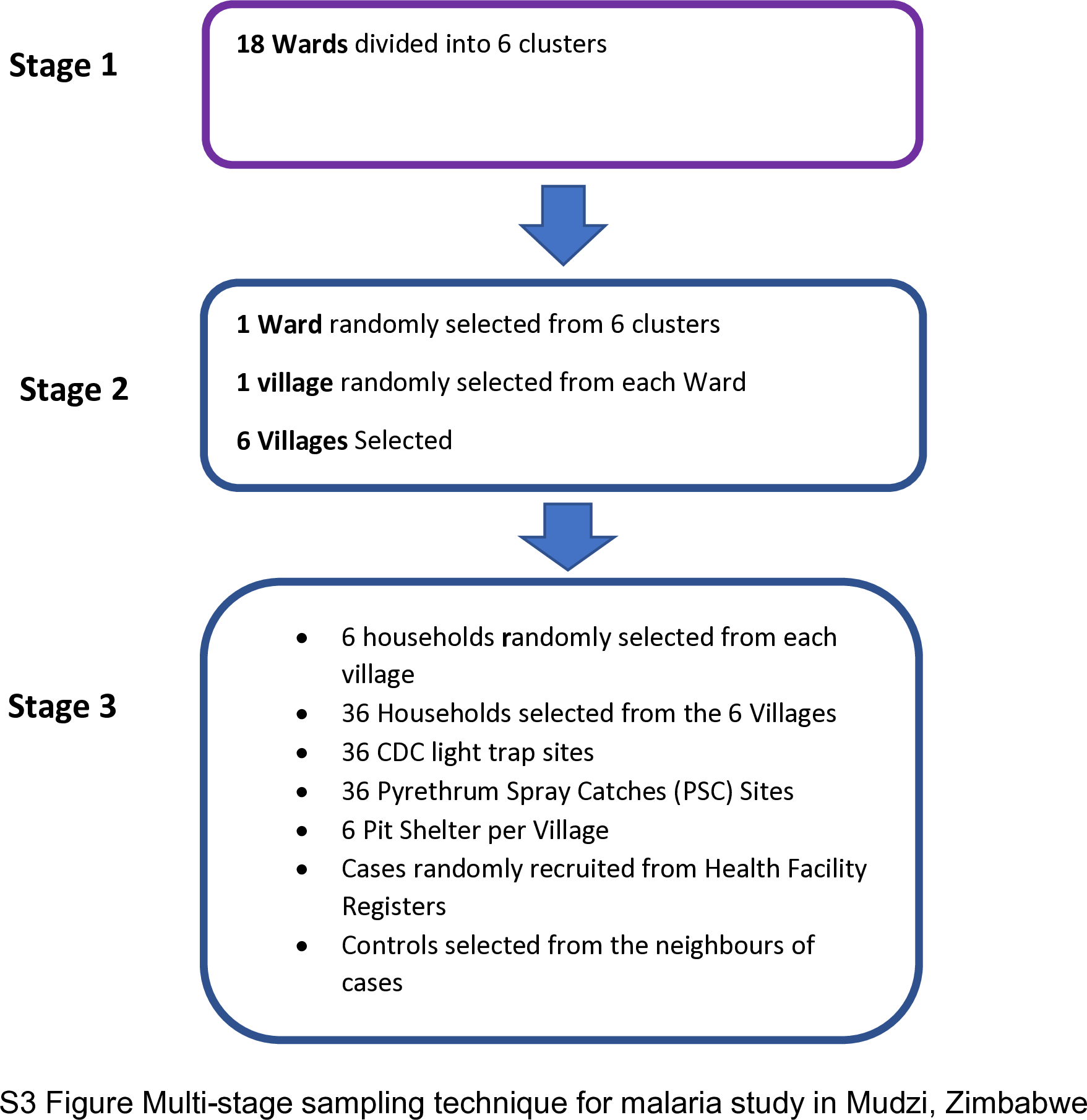

Supplement: S3 Fig — (TIF) [file pone.0329093.s003.tif]
