# Supplementary material for: Risk factors for contracting malaria in six wards of Mudzi District, Zimbabwe: A case control-study
Source: PLoS One. 2025 Aug 7;20(8):e0329093. doi: 10.1371/journal.pone.0329093 (PMC12331041; doi:10.1371/journal.pone.0329093)
Supplement: Attachment — Submitted filename: Manuscript Number PONE-D-24-44171_for author.docx [file pone.0329093.s004.docx]

**Reviewer Recommendation and Comments for Manuscript Number PONE-D-24-44171**

**Major Comments**

1. **Research Gap and Study Objective (Lines 11–17):**
   - The abstract begins with the global impact of malaria and provides context for the study's importance. However, despite the well-performing IRS, the research team highlights the persistence of malaria transmission in Mudzi. For example:
     *"Despite significant reductions in the malaria burden in Zimbabwe, persistent high transmission in districts like Mudzi indicates a potential role of behavioral and environmental factors unaddressed by current interventions."*
   - Clearly articulate how the study addresses a novel aspect or adds to existing literature.
2. **Study Design and Methods (Lines 17–23):**
   - The methods are well-described, but the recruitment process for cases and controls needs clarification. Specifically:
     - Were controls matched in any way beyond being neighbours? If not, why was an unmatched design chosen?
     - Specify the criteria for classifying a participant as a "case" beyond malaria facility registers.
   - The use of the Kobo Collect tool is noted but lacks justification. Add a brief rationale for its use (e.g., efficiency, accuracy, or real-time data collection in rural settings).
3. **Key Findings and Interpretation (Lines 24–28):**
   - The findings are clearly reported with adjusted odds ratios (AOR) and confidence intervals. However:
     - The abstract lists the results but does not provide sufficient interpretation. For example, why might outdoor night activities (religious and social) and gardening increase risk? How do these findings align with or differ from other studies?
     - Briefly address why knowledge about malaria (high in both groups) did not translate to reduced risk for cases.
4. **Conclusions and Recommendations (Lines 31–33):**
   - The conclusion emphasizes behavior change strategies to complement IRS but lacks specific recommendations. Strengthen this by suggesting actionable measures, such as targeted educational campaigns or community-based interventions during high-risk periods (e.g., night religious gatherings).

**Major Comments**

1. **Clarity and Presentation of Demographics (Lines 193–201):**
   - The demographic results mentioned are precise but could be more concise and reader-friendly. For example, instead of stating, "There were no significant differences (p>0.05) between cases and controls on demographic variables," consider rephrasing to group insights, such as:
     *"Age, marital status, employment status, and income distribution were comparable between cases and controls, with no statistically significant differences observed (p>0.05)."*
   - Ensure the comparisons align with the data shown in Table 1 and highlight any relevant demographic findings.
2. **Sampling and Study Design (Lines 122–139):**
   - The explanation of sampling is detailed but lacks clarity in connecting the rationale for multi-stage sampling to the study objectives. Consider explicitly justifying the choice of six wards and how they adequately represent the district.
   - It might help to explain why neighbours of cases were chosen as controls and how potential confounding factors, such as shared environmental risks, were accounted for.
3. **Key Informant Interviews (Lines 157–164):**
   - The role of the key informant interviews in complementing the case-control study is not clearly explained. What specific insights or triangulation did the interviews add to the findings? A clear link between these data and the overall research objectives would enhance the manuscript.
4. **Data Analysis (Lines 166–179):**
   - The criteria for selecting variables with a p-value of 0.25 for logistic regression should be briefly justified, as this threshold may not be intuitive to all readers.
   - The Shapiro-Wilk test is used for normality testing, but it would help to mention whether data transformations were applied for non-normal data or how this affected subsequent analyses.
5. **Knowledge Scoring (Lines 180–181):**
   - The method for assessing malaria knowledge is straightforward but would benefit from additional context. How were these knowledge thresholds determined? Were they adapted from a prior study or guideline? Clarify how knowledge was assessed across the case and control groups.
6. **Ethics and Approvals (Lines 183–190):**
   - Researchers ensured participant confidentiality during the physical follow-ups in villages, but the methods used to maintain it are not mentioned in the ethical considerations. Adding a brief note on this aspect would strengthen the ethical rigor of the manuscript.

**Major Comments**

1. **Clarity and Presentation of Demographics:**
   - The authors can improve the narrative describing the findings (lines 193–201) to enhance the demographic results' readability in Table 1. For example, the statement "There were no significant differences (p>0.05) between cases and controls on demographic variables" could be supported with more concise explanations or grouped insights, such as by stating, "Age, marital status, employment status, and income distribution were comparable between cases and controls."
2. **Risk Factors:**
   - The analysis of risk factors (lines 204–219) is valuable, but there is some redundancy in presenting odds ratios in both text and tables. Consider summarizing only the most significant findings in the text (e.g., wearing protective clothing, sleeping in sprayed rooms, and attending church services) while directing readers to the table for full details.
   - The multivariate analysis (Table 3) is compelling, but the forward stepwise method used for model building lacks detail. Explain the criteria used to include or exclude variables in the model, as this is critical to assess the robustness of the findings.
3. **Knowledge of Malaria Transmission:**
   - The authors could further discuss the statistical significance of differences in prevention actions (p=0.05) and perceptions of chemical efficacy (p<0.001) to explore their practical implications for public health interventions . For example, how can this information be leveraged to enhance malaria prevention strategies?
4. **Health Service Delivery:**
   - The section on health service delivery (lines 231–233) is underdeveloped. While it is stated that most participants felt they received good treatment, the specific measures used to assess "good treatment" or "friendly staff" are unclear. Elaborate on how these perceptions were measured (e.g., through structured interviews or surveys) and consider the potential for recall bias in self-reported data.
5. **Statistical Reporting and Interpretation:**
   - The presentation of p-values and confidence intervals is appropriate; however, the manuscript lacks a discussion on potential confounding factors that may affect the observed associations. For example, were socioeconomic factors or healthcare access considered potential confounders?
   - In Table 3, the AOR for attending morning and night church services (8.13; 95% CI: 1.74–37.90) has a wide confidence interval, suggesting limited precision. This warrants further discussion regarding potential issues such as sample size or variability in this subgroup.
6. **Ethical Considerations and Study Limitations:**
   - There is no mention of ethical approval or participant consent in the provided text. Ensure this information is clearly stated in the manuscript.
   - The authors should acknowledge the study's limitations, including reliance on self-reported behaviors and potential selection bias in recruiting cases and controls. They should also discuss the generalizability of the findings to other populations or regions

**Minor Comments**

1. **Terminology Consistency (Lines 15–17):**
   - Replace "well-performing indoor residual spraying program" with more precise language, such as "effective indoor residual spraying (IRS) program," to avoid redundancy.
2. **Statistical Analysis Details (Lines 23–24):**
   - Replace "stepwise forward multivariate logistic regression analysis" with a more concise phrase, such as "multivariable logistic regression analysis." Avoid overly technical jargon in the abstract.
3. **Protective Factors (Lines 27–28):**
   - The phrase "putting on long clothes that cover the whole body at night" is wordy. Simplify to "wearing full-body clothing at night."
4. **Knowledge Assessment Results (Lines 28–30):**
   - The phrase "knew at least one major method of controlling malaria" is vague. Specify what constitutes a "major method" (e.g., use of mosquito nets, IRS).
5. **Key Drivers of Malaria Transmission (Lines 31–32):**
   - Replace "key drivers of malaria transmission" with "primary risk factors for malaria transmission" to align with scientific terminology.
6. **Keywords (Lines 34):**
   - The keywords are relevant but could be expanded to include "Indoor Residual Spraying," "Behavior Change," and "Case-Control Study" to improve discoverability.

**Minor Comments**

1. **Terminology Consistency (Lines 93–95):**
   - The manuscript switches between "RDT test" and "positive RDT test result." For consistency, use a single term like "positive RDT result."
2. **Geographic Description (Lines 98–104):**
   - The description of Mudzi district could benefit from a more concise summary. For example, "Mudzi, located on the Zimbabwe-Mozambique border, has 21 administrative wards serving a population of approximately 165,266 (2022 Census)."
3. **Use of Abbreviations (Lines 104–106):**
   - Avoid overloading the reader with abbreviations in a single sentence (e.g., RMNCH, EPI, HIV & TB). Consider explaining only the most relevant terms in the main text and moving others to a footnote or supplementary material.
4. **Tool Translation (Lines 155–156):**
   - While the translation of tools into Shona is commendable, you should specify how translation accuracy and consistency were ensured (e.g., through back-translation or pre-testing).
5. **Sampling Tool Reference (Lines 126–127):**
   - The inclusion of "[www.randomlist.com](http://www.randomlist.com/)" in the text might be better placed in a footnote or omitted to maintain a formal tone.
6. **Figure 1 Placement (Lines 99–129):**
   - The reference to Figure 1 is ambiguous. Ensure the figure is placed appropriately within the manuscript and fully described in the caption.
7. **Data Cleaning Description (Lines 167–169):**
   - While you describe data cleaning methods like scatter plots and frequency tables, it may help to briefly state what common outliers were found (if any) and how they were handled.
8. **Typographical Issues:**
   - Line 104: Replace "25 )(39)" with a clearer reference format.
   - Line 119: Clarify whether the total sample size was 132 or 66 cases and 66 controls. Avoid redundancy.

**Minor Comments**

1. **Grammar and Syntax:**
   - Lines 194–195: Replace "94 [50.81%] cases and 91[49.19%]" with "94 (50.81%) cases and 91 (49.19%) controls" for consistency.
   - Line 223: "General body weakness (cases – 52.13%, controls – 48.35%) and loss of appetite..." could be simplified to "General body weakness (52.13% of cases and 48.35% of controls) and loss of appetite..."
2. **Table Presentation:**
   - In Table 1, ensure consistent formatting for percentages and alignment of columns. For instance, percentages should be rounded to one or two decimal places for clarity (e.g., 73.12% and 74.44%).
   - Tables 2 and 3 should include clear captions explaining the analysed variables, methods, and statistical tests.
3. **Terminology:**
   - Avoid ambiguous terms such as "good treatment" (line 231). Specify criteria or metrics used for this assessment.
   - Replace "the odds of suffering from malaria" (line 216) with "the odds of contracting malaria" for accuracy.
4. **Formatting:**
   - Ensure tables and figures follow PLoS ONE formatting guidelines, including appropriate captions and footnotes for statistical results.
   - The text should use a consistent style for referencing p-values (e.g., p = 0.05 instead of p=0.05).

**Reporting Guidelines (STROBE)**

Following the STROBE guidelines (Strengthening the Reporting of Observational Studies in Epidemiology) is essential for observational studies like this. The manuscript does a good job of detailing the study design, sampling, and data collection procedures. However, make sure that all relevant STROBE items are addressed, including:

- **Participant flow**: Were any cases or controls excluded after enrollment? This should be clearly stated.
- **Confounding variables**: Were potential confounders measured, and how were they controlled for in analysis?
- **Outcome measures**: Clearly define the primary and secondary outcome measures and describe how they were measured.

Explicitly mention **participant flow**, including the number of cases/controls excluded and why, and confirm how confounders were addressed in the analysis.Ensure that the abstract captures the novelty of the research and emphasizes how the findings can inform public health strategies.

**Recommendations for Revision**

1. Clarify the methodology for statistical analysis, particularly the criteria for including variables in the multivariate model.
2. Expand the discussion of significant findings, linking them to potential public health interventions or implications.
3. Address limitations and potential biases more explicitly, as well as their impact on the study's conclusions.
4. Provide details on ethical approval, participant recruitment, and informed consent to strengthen the study's credibility.
5. Improve table formatting and streamline the narrative to avoid redundancy between text and tables.

The manuscript is well-structured and provides detailed methodology. However, the narrative can benefit from clearer explanations and reduced redundancy in sections like demographics, sampling, and data collection. Ensure that all abbreviations are consistently used and explained. Strengthen the link between the qualitative data from key informants and the quantitative results, highlighting how this adds depth to the analysis.
