# Supplementary material for: Risk factors for contracting malaria in six wards of Mudzi District, Zimbabwe: A case control-study
Source: PLoS One. 2025 Aug 7;20(8):e0329093. doi: 10.1371/journal.pone.0329093 (PMC12331041; doi:10.1371/journal.pone.0329093)
Supplement: Attachment — Submitted filename: Response to Reviewers.docx [file pone.0329093.s007.docx]

| **REVIEWER #1** | **REVIEWER COMMENT** | **AUTHOR COMMENTS** | **Changed section** |
| --- | --- | --- | --- |
|  | 1. Research Gap and Study Objective (Lines 11–17): The abstract begins with the global impact of malaria and provides context for the study's importance. However, despite the well-performing IRS, the research team highlights the persistence of malaria transmission in Mudzi. For example: "Despite significant reductions in the malaria burden in Zimbabwe, persistent high transmission in districts like Mudzi indicates a potential role of behavioral and environmental factors unaddressed by current interventions" o Clearly articulate how the study addresses a novel aspect or adds to existing literature. | The justification has been modified to read as follows  Despite a well-performing IRS, the persistent malaria transmission in the district reveals the critical role of vectoral behavior, human behavior, and health system factors which have not been fully investigated. This case-control nested in a larger study, was conducted to determine the risk factors responsible for persistent malaria occurrence in the district | Line 17-20 |
|  | 2. Study Design and Methods (Lines 17–23): o The methods are well-described, but the recruitment process for cases and controls needs clarification. Specifically: | Cases were randomly recruited from malaria facility registers, whilst controls were recruited from a village household register. A case was defined as an individual residing in a selected village who tested malaria-positive by RDT. A control was an individual with no recorded positive RDT result in the facility or community health worker malaria register | Line 23-27 |
|  | Were the controls matched in any way beyond being neighbours? If not, why was an unmatched design chosen? | Controls were not matched in any way beyond being neighbours. An unmatched design was used due to the likelihood of differences in characteristics of the households, including preventive, socio-economic characteristics. |  |
|  | Specify the criteria for classifying a participant as a "case" beyond malaria facility registers. The use of the Kobo Collect tool is noted but lacks justification. Add a brief rationale for its use (e.g., efficiency, accuracy, or real-time data collection in rural settings). | A case was defined as an RDT-positive individual from facility registers residing in a selected village.  The justification for the use of Kobo Collect has been inserted: Kobo Collect was used for real-time data collection……. | Line24-27  Lines 30-31 |
|  | 3. Key Findings and Interpretation (Lines 24–28): The findings are reported with adjusted odds ratios (AOR) and confidence intervals. However: The abstract lists the results but does not provide sufficient interpretation. For example, why might outdoor night activities (religious and social) and gardening increase risk? How do these findings align with or differ from other studies? | A brief interpretation of the results has been provided in the revised abstract  Higher night outdoor activity for social and religious purposes increases the exposure of people to mosquito bites  The comparability and contrast of our study to other studies have been included in the main manuscript discussion section | Lines 38-41  Line 332-340  359-371 |
|  | Briefly address why knowledge about malaria (high in both groups) did not translate to reduced risk for cases. | An explanation of why high knowledge did not translate to protective behavior is provided in the revised discussion section:  Key determinants cited in the section:   - Unemployment is pushing local communities to engage in artisanal gold mining for survival. - Migratory farming practices - Social needs (religion and entertainment) - Cross-border movement for trading purposes and connecting with extended family or social connections across borders | Lines 37-47 and 372-386 |
|  | 4. Conclusions and Recommendations (Lines 31–33): o The conclusion emphasizes behavior change strategies to complement IRS but lacks specific recommendations. Strengthen this by suggesting actionable measures, such as targeted educational campaigns or community-based interventions during high-risk periods (e.g., night religious gatherings). | Detailed recommendations have also been provided in the main text  A brief, compressed, actionable recommendation is given in the abstract:  Sustaining IRS and intensifying, integrated, targeted community engagement and malaria awareness programs will be key in eliminating malaria in Mudzi | Line 41-44 |
|  | 1. Clarity and Presentation of Demographics (Lines 193–201): The demographic results mentioned are precise but could be more concise and reader-friendly. For example, instead of stating, "There were no significant differences (p>0.05) between cases and controls on demographic variables," consider rephrasing to group insights, such as: "Age, marital status, employment status, and income distribution were comparable between cases and controls, with no statistically significant differences observed (p>0.05)." o Ensure the comparisons align with the data shown in Table 1 and highlight any relevant demographic findings. | Thank you for this valuable suggestion.  The description of Table 1 has been modified as recommended to read:  “Case and controls were comparable on demographic variables such as age, marital status, employment status, and income distribution, with no statistically significant differences observed (p>0.05), T**able 1.** | Table 1  Line 237-242 |
|  | 2. Sampling and Study Design (Lines 122–139): The explanation of sampling is detailed but lacks clarity in connecting the rationale for multi-stage sampling to the study objectives. Consider explicitly justifying the choice of six wards and how they adequately represent the district. | The comment is well appreciated. Further clarification of how the six villages were selected has been provided.  Since the study is nested in a larger study further details have also been provided on the primary study sampling design, which informed the current study. Additional files have also been added to clarify the sampling design. **Supplementary file 1** and **Fig 1** has been provided to give clarity on the geospatial distribution of the selected sites. | Lines 128-141 |
|  | It might help to explain why neighbors of cases were chosen as controls and how potential confounding factors, such as shared environmental risks, were accounted for. | Neighbors were chosen from the same village to allow for comparability.  Control households were within the same environment and had similar socio-behavioural aspects, but with no infected individual.  Logistical reasons and cost also informed the selection of the control from the case neighborhood  Possible known confounders, such as differences in Age, Sex, housing structure, and access to a community health worker, were accounted for in the MVLA. |  |
|  | 3. Key Informant Interviews (Lines 157–164): The role of the key informant interviews in complementing the case-control study is not clearly explained. What specific insights or triangulation did the interviews add to the findings? A clear link between these data and the overall research objectives would enhance the manuscript. | The comment is well appreciated.  Additional justification for Key informant interviews has been added to the text.  “Key informant interviews (KI) were also conducted to provide additional qualitative perspectives on malaria transmission drivers, primary community socio-economic behaviour, and health system capacities.:” | Line 180-189 |
|  | 4. Data Analysis (Lines 166–179): The criteria for selecting variables with a p-value of 0.25 for logistic regression should be briefly justified, as this threshold may not be intuitive to all readers. The Shapiro-Wilk test is used for normality testing, but it would help to mention whether data transformations were applied for non-normal data or how this affected subsequent analyses. | No data transformations were done. Mean and standard deviations were used for normally distributed data, and median and interquartile range were used for data deviating from normality. |  |
|  | 5. Knowledge Scoring (Lines 180–181): The method for assessing malaria knowledge is straightforward but would benefit from additional context. How were these knowledge thresholds determined? Were they adapted from a prior study or guideline? Clarify how knowledge was assessed across the case and control groups. | Your comment is highly appreciated. We have provided additional information on how knowledge was assessed in this study.  Knowledge was assessed using the modified Bloom’s total knowledge and attitude cut-off points as shown in the revised text  A modified Bloom cut-off for total knowledge and attitude was used to assess knowledge of malaria using proportions scores methods described by Lopez et al.(42) and Okello et al. (43) where a score of 80-100% is good knowledge, 60-79 is moderate knowledge, and <59% is poor knowledge. | Line 212-215 |
|  | 6. Ethics and Approvals (Lines 183–190): Researchers ensured participant confidentiality during the physical follow-ups in villages, but the methods used to maintain it are not mentioned in the ethical considerations. Adding a brief note on this aspect would strengthen the ethical rigor of the manuscript. | Additional information on how confidentiality was maintained during and after the study has been added to the text.  Access to the Kobo Collect storage was restricted to the researchers with usernames and passwords.  The collected information was not transferable.  After submission of each electronic questionnaire, the data was not retained on the individual phone or tablet.  Personal data was only collected on consent forms for Ethical review and compliance purposes only. The data was not included in any analysis. | Lines 223-229 |
|  | Major Comments 1. Clarity and Presentation of Demographics: The authors can improve the narrative describing the findings (lines 193–201) to enhance the demographic results' readability in Table 1. For example, the statement "There were no significant differences (p>0.05) between cases and controls on demographic variables" could be supported with more concise explanations or grouped insights, such as by stating, "Age, marital status, employment status, and income distribution were comparable between cases and controls." | This observation is highly appreciated. The necessary adjustments have been made to the description of demographics. Case and controls were comparable on demographic variables such as age, marital status, employment status, and income distribution with no statistically significant differences observed (p>0.05), T**able 1.** There was no significant difference between cases and controls in terms of gender, education, marital status, employment religion, and income status | Line 232-237 |
|  | 2. Risk Factors: The analysis of risk factors (lines 204–219) is valuable, but there is some redundancy in presenting odds ratios in both text and tables. Consider summarizing only the most significant findings in the text (e.g., wearing protective clothing, sleeping in sprayed rooms, and attending church services) while directing readers to the table for full details. The multivariate analysis (Table 3) is compelling, but the forward stepwise method used for model building lacks detail. Explain the criteria used to include or exclude variables in the model, as this is critical to assess the robustness of the findings. | The comment is well noted. A revision has been done in the manuscript to reduce redundancy  The methods section has been expanded to give more detail on how variables were included and excluded in the Multivariate model.  The p=0.25 cut-off is based on the Hosmer and Lemeshow's purposeful selection of covariates, whereby in the univariate analysis, any variable having a significant univariate test at some arbitrary level (0.25 in our case) is selected as a candidate for the multivariate analysis. | Line 200-211 |
|  | 3. Knowledge of Malaria Transmission: The authors could further discuss the statistical significance of differences in prevention actions (p=0.05) and perceptions of chemical efficacy (p<0.001) to explore their practical implications for public health interventions. For example, how can this information be leveraged to enhance malaria prevention strategies? | Implications of intervention and remedial actions are fully discussed in the revised text  Differences in perception of efficacy and knowledge of preventative measures within the population will result in differential uptake | Line 426-432 |
|  | 4. Health Service Delivery: The section on health service delivery (lines 231–233) is underdeveloped. While it is stated that most participants felt they received good treatment, the specific measures used to assess "good treatment" or "friendly staff" are unclear. Elaborate on how these perceptions were measured (e.g., through structured interviews or surveys) and consider the potential for recall bias in self-reported data. | The assessment of perceptions and attitudes toward malaria treatment services was conducted using a structured questionnaire.  The possible likelihood of recall bias occurrence in this case study is noted and acknowledged in the limitations section. | Line 25-28 |
|  | 5. Statistical Reporting and Interpretation: The presentation of p-values and confidence intervals is appropriate; however, the manuscript lacks a discussion on potential confounding factors that may affect the observed associations. For example, were socioeconomic factors or healthcare access considered potential confounders? | This observation is highly appreciated  The possible confounders  ( age, sex, habitation status, access to community health care workers, and net ownership ) were considered in the Multi variable logistic regression analysis | Line 244-249 |
|  | In Table 3, the AOR for attending morning and night church services (8.13; 95% CI: 1.74–37.90) has a wide confidence interval, suggesting limited precision. This warrants further discussion regarding potential issues such as sample size or variability in this subgroup. | The observation is appreciated. The observed wide confidence interval could be due to the sample size for the particular variable. |  |
|  | 6. Ethical Considerations and Study Limitations: There is no mention of ethical approval or participant consent in the provided text. Ensure this information is clearly stated in the manuscript. The authors should acknowledge the study's limitations, including reliance on self-reported behaviors and potential selection bias in recruiting cases and controls. They should also discuss the generalizability of the findings to other populations or regions | The ethical statement has been provided in the text  The limitation section has been reviewed to incorporate these valuable comments. Recall bias and the likelihood of misclassification bias of cases and controls, and implications are explained in the revised limitation sections. | Lines 204-217 and Lines 374-379. |
|  | **Minor Comments** |  |  |
|  | 2. Statistical Analysis Details (Lines 23–24):  Replace "stepwise forward multivariate logistic regression analysis" with a more concise phrase, such as "multivariable logistic regression analysis." Avoid overly technical jargon in the abstract. | The comment is highly appreciated.  The statement has been amended in the abstract. | Line 32 |
|  | 1. Terminology Consistency (Lines 15–17): o Replace "well-performing indoor residual spraying program" with more precise language, such as "effective indoor residual spraying (IRS) program," to avoid redundancy. | The statement has been revised to read “Despite an high IRS coverages in the district…” | Line 17 |
|  | 4. Knowledge Assessment Results (Lines 28–30): The phrase "knew at least one major method of controlling malaria" is vague. Specify what constitutes a "major method" (e.g., use of mosquito nets, IRS). | The statement has been revised to read as follows:  The majority of cases (96.74%) and controls (92.22%) had good knowledge of malaria transmission and preventative measures | Line 37-40 |
|  | 3. Protective Factors (Lines 27–28): o The phrase "putting on long clothes that cover the whole body at night" is wordy. Simplify to "wearing full-body clothing at night." | The recommended wording has been adopted,  The statement has been modified to read  “Wearing full body clothing at night” | Line 34 |
|  | 5. Key Drivers of Malaria Transmission (Lines 31–32): o Replace "key drivers of malaria transmission" with "primary risk factors for malaria transmission" to align with scientific terminology. | The text has been revised as suggested. It now reads:  We therefore conducted this unmatched case-control study to identify human socio-economic behavioural and health system primary factors responsible for persistent malaria transmission in the district | Line 18-21 |
|  | 6. Keywords (Lines 34): The keywords are relevant but could be expanded to include "Indoor Residual Spraying," "Behavior Change," and "Case-Control Study" to improve discoverability. | The suggested additional words have been added to the keywords. | Lines 42-43 |
|  | Minor Comments |  |  |
|  | 1. Terminology Consistency (Lines 93–95): The manuscript switches between "RDT test" and "positive RDT test result." For consistency, use a single term like "positive RDT result." | The suggested term has been adopted, and “a **positive RDT result”** is now the adopted term throughout the whole text. |  |
|  | 2. Geographic Description (Lines 98–104): The description of Mudzi district could benefit from a more concise summary. For example, "Mudzi, located on the Zimbabwe-Mozambique border, has 21 administrative wards serving a population of approximately 165,266 (2022 Census)." | The statement on Mudzi has been reviewed to read as follows:  “Mudzi (Geo-coordinates: 17˚00ʹS032˚40ʹE) is one of the nine (9) administrative districts in Mashonaland East Province, located on the eastern border region of Zimbabwe and Mozambique with a population of 165,266.” | Line 118-112 |
|  | 3. Use of Abbreviations (Lines 104–106): o Avoid overloading the reader with abbreviations in a single sentence (e.g., RMNCH, EPI, HIV & TB). Consider explaining only the most relevant terms in the main text and moving others to a footnote or supplementary material. | The extra abbreviations have been removed from the text. The statement now reads as follows:  “Each administrative ward is served by a single public health facility, Rural Health Center (RHC), which offers an array of essential primary health care services packages ranging from maternal child health, malaria treatment, immunization, and HIV and TB.” | Line 114-116 |
|  | 4. Tool Translation (Lines 155–156): o While the translation of tools into Shona is commendable, you should specify how translation accuracy and consistency were ensured (e.g., through back-translation or pre-testing). | Additional details have been added to the methods section  The questionnaire was back-translated by two independent individuals from English to Shona and back to English. The translation was checked for consistency. Pre-testing of the tool was conducted in one of the target villages. Vague, ambiguous, and difficult questions were reviewed and revised. | Line 171-179 |
|  | 5. Sampling Tool Reference (Lines 126–127): The inclusion of "[www.randomlist.com](https://url.za.m.mimecastprotect.com/s/dnvJCRgBjWSY6n0DIjtZT14vhx?domain=randomlist.com)" in the text might be better placed in a footnote or omitted to maintain a formal tone. | The recommendation has been adopted, and the website now appears in the footnote | Line 142 Pg 7 |
|  | Minor Comments  6. Figure 1 Placement (Lines 99–129): | **Figure 1** has been placed in the text | Line 144 |
|  | The reference to Figure 1 is ambiguous. Ensure the figure is placed appropriately within the manuscript and fully described in the caption. | **Figure 1** has been inserted into the manuscript text | Line 144 |
|  | 7. Data Cleaning Description (Lines 167–169):  While you describe data cleaning methods like scatter plots and frequency tables, it may help to briefly state what common outliers were found (if any) and how they were handled. | No data outliers were found during data inspection. |  |
|  | 8. Typographical Issues: o Line 104: Replace "25 )(39)" with a clearer reference format. o Line 119: Clarify whether the total sample size was 132 or 66 cases and 66 controls. Avoid redundancy. | The typo has been rectified as follows  “The calculated total sample size was 66 cases and 66 controls.” | Line 124 |
|  | Minor Comments 1. Grammar and Syntax: o Lines 194–195: Replace "94 [50.81%] cases and 91[49.19%]" with "94 (50.81%) cases and 91 (49.19%) controls" for consistency. | Corrections on grammar and syntax have been made as recommended in the text | Lines 232 |
|  | Line 223: "General body weakness (cases – 52.13%, controls – 48.35%) and loss of appetite..." could be simplified to "General body weakness (52.13% of cases and 48.35% of controls) and loss of appetite..." | We appreciate the suggestion. The statement has been amended and now appears as  Knowledge of malaria transmission was high among cases and controls, with 96.74% of cases and 92.22% of controls being aware of what causes malaria. General body weakness ( 52.13% of cases and 48.35% of controls) and loss of appetite (11.7% of cases and 13.19% of controls) were the two most reported signs and symptoms of malaria identified, as depicted in Table 4 | Line: 293-296 |
|  | 2. Table Presentation:   In Table 1, ensure consistent formatting for percentages and alignment of columns. For instance, percentages should be rounded to one or two decimal places for clarity (e.g., 73.12% and 74.44%). Tables 2 and 3 should include clear captions explaining the analyzed variables, methods, and statistical tests. | The percentages in the tables have been rounded to two decimal places.  The captions for Tables 2 and 3 have been improved to show the variables analyzed and the statistical test conducted. |  |
|  | 3. Terminology: Avoid ambiguous terms such as "good treatment" (line 231). Specify the criteria or metrics used for this assessment. | The text has been revised as shown:  There was no significant difference between the views on quality of service at health facilities between cases and controls (p>0.05). The majority of cases (71.11%) and controls (70.00%) were of the view that the quality of service offered at health facilities was good Table 5. | Lines 304-306 |
|  | Replace "the odds of suffering from malaria" (line 216) with "the odds of contracting malaria" for accuracy. | This section has been revised | Line 285-288 |
|  | 4. Formatting: o Ensure tables and figures follow PLoS ONE formatting guidelines, including appropriate captions and footnotes for statistical results. | The captions for the Tables have been improved to align with PLOS ONE formatting guidelines. |  |
|  | The text should use a consistent style for referencing p-values (e.g., p = 0.05 instead of p=0.05). Reporting Guidelines (STROBE) | The observation is well appreciated  The p-value referencing has been revised in the text  p = 0.05 has been removed and replaced with p<0.05 | Line 260 |
|  | Following the STROBE guidelines (Strengthening the Reporting of Observational Studies in Epidemiology) is essential for observational studies like this. The manuscript does a good job of detailing the study design, sampling, and data collection procedures. However, make sure that all relevant STROBE items are addressed, including: • Participant flow: Were any cases or controls excluded after enrollment? This should be clearly stated. • Confounding variables: Were potential confounders measured, and how were they controlled for in analysis? | Potential confounding factors were controlled for at analysis, whereby they were included in the regression model alongside the primary independent variables of interest. The regression coefficient will then reflect the adjusted relationship between the primary independent variable and the dependent variable, hence, adjusted odds ratios were reported | Line 202-210  Line 289 |
|  | • Outcome measures: Clearly define the primary and secondary outcome measures and describe how they were measured. Explicitly mention participant flow, including the number of cases/controls excluded and why, and confirm how confounders were addressed in the analysis. Ensure that the abstract captures the novelty of the research and emphasizes how the findings can inform public health strategies. | The primary outcome measures (OR and AOR) have been fully described in the methods section and data analysis section.  The MVLA methods of selecting eligible variables into the model have been fully explained in the methods section | Line 202-213 |
|  | **Recommendations for Revision** |  |  |
|  | 1. Clarify the methodology for statistical analysis, particularly the criteria for including variables in the multivariate model. | A full explanation on the inclusion criteria for variables in the multivariate model has been outlined.  **Criteria Rationale:**  Using a higher cut-off p-value as a criterion for including variables in the multivariate model is based on the Hosmer and Lemeshow purposeful selection of covariates, whereby in the univariate analysis, any variable having a significant univariate test at some arbitrary level (0.25 in our case) is selected as a candidate for the multivariate analysis. This is based on the Wald test from logistic regression and a p-value cut-off of 0.25*.* | Lines 202-212 |
|  | 2. Expand the discussion of significant findings, linking them to potential public health interventions or implications. | The discussion section has been expanded, linking major findings to potential public health interventions  The new expansion is highlighted in the new text | Line 358-453 |
|  | 3. Address limitations and potential biases more explicitly, as well as their impact on the study's conclusions. | Limitations have been fully addressed and impacts explained. | Line 444-452 |
|  | 4. Provide details on ethical approval, participant recruitment, and informed consent to strengthen the study's credibility. | Additional details on ethical approval have been provided | Lines 217-229 |
|  | 5. Improve table formatting and streamline the narrative to avoid redundancy between text and tables. | Tables 1,2,3,4 have been formatted  Redundancy has been reduced in the text, especially text connecting to Tables 1,2,3 |  |
|  | The manuscript is well-structured and provides a detailed methodology. However, the narrative can benefit from clearer explanations and reduced redundancy in sections like demographics, sampling, and data collection. Ensure that all abbreviations are consistently used and explained. Strengthen the link between the qualitative data from key informants and the quantitative results, highlighting how this adds depth to the analysis. | The link between qualitative and Quantitative has been incorporated into the revised discussion section, showing the impact of KI data and quantitative results  The methods and discussion have been revised to clearly outline the rationale for KI  In the discussion section, we have highlighted the key finding from KI interviews findings in terms of outdoor movement, health care worker coverage, community religious, social, farming and occupational practices and their effect on IRS. A link between the qualitative results and quantitative results has also been established in the discussion text. | Line 385-425 |
|  | **REVIEWER # 2** |  |  |
|  | Thanks for the opportunity to review this paper on an important public health topic. I have the following comments: 1. There is a missing word after "reduction in financial..." in line 46. | The missing word has been inserted  The sentence now reads as:  “reduction in malaria control financial programmatic support.” | Line 56 |
|  | 2. In line 50, I propose the authors just say children as opposed to saying "children under 5 years and over 5 years..." | The recommendation is well appreciated.  The text has been revised to read as recommended*: “*Pregnant women and children are …….” | Line 60 |
|  | 3. Line 83, the authors undertook this study in "five wards," which contradicts what is in the title and abstract | The typo has been rectified to read six (6) wards, | Line 63 |
|  | 4. Line 86, there is a missing word after "in one rural health...". Is this a center or a clinic? | In Zimbabwe, the official title for a rural health facility/clinic is  “Rural health center”-RHC  This term is maintained throughout the text | Line 96 |
|  | 5. It would be interesting to know why controls were recruited from neighbors when they could have been recruited from negative individuals in the RDT register and, in so doing, offset the chances of misclassification of controls | A single rural health center serves an administrative ward. A ward is composed of 5-15 villages. A village is composed of 10 to over 100 households, depending on geographic location.  Recruiting cases from the same village as controls ensured that controls inherently had the same exposure of interest as cases except that they didn’t have malaria illness.  This recruitment was influenced by the critical requirement that controls should be comparable to the source population of cases as possible.  Lastly recruiting controls from the same village as cases posed minimal logistic and financial challenges since the 6 selected villages in the study were separated by great distances as shown on Fig 1.  Likelihood of occurrence of misclassification bias is real. This limitation is acknowledged in the limitation of study section | Line 154-159 |
|  | 6. Were the study participants all adults, or did the sample include both adults and children? This is not clear. | The study included minors and adults.  The study recruited minors (aged <18 years) shown in Table 1. | Line 224 |
|  | 7. Under eligibility, the exclusion criteria again refer to five wards. Kindly correct this. | The typo has been corrected to read  Six (6) villages. | Line 161 |
|  | 8. The authors also mention individuals who did not give verbal or written consent. What form of consent was approved by the IRB when the protocol was approved? It is not standard practice to have others consent verbally while others are offered written consent in the same study | For this study, informed written consent was utilised  The consent forms were both in the local language and English. The consent forms in English and the Local language were also approved by BREC and MRCZ  As shown in the text Informed written consent was obtained from all participants. Only those who consented by signing the consent form were recruited into the study. For minors- those aged below the age of 18 years, written parental /guardian consent was obtained for all the participants. | Lines 206-211 |
|  | 9. Where were the interviews conducted? How was confidentiality assured? | Cases and control were interviewed at their homesteads, while key informants were interviewed at their workplaces.  Data was collected through Kobo Collect forms on phones. After submission to the Kobo Collect server, the submitted forms were not retained on the phone. Only the researchers TF, PN, and MJC had usernames and passwords to access collected data | Line 189-190  Line 212-223 |
|  | 10. How did the researchers ensure that all research assistants asked the questions the same way during interviews? There is a high chance that the interpretation of English to the local language at interviewing participants might have introduced an error | Training of Research Assistants was conducted to ensure they understood the objectives of the paper and the questionnaires.  Pre-testing of the tool was conducted in one of the selected villages to familiarize the RAs with the tool and to identify and correct any unclear, ambiguous, and difficult questions.  A back translation of the questionnaire was also conducted to ensure it accurately captured the intents of the English version.  This is outlined in the revised methods section. | Line 177-180 |
|  | 11. Capitalize the "d" in the title "district Medical Officer". Insert commas where appropriate is a number of sentences. | The word has been capitalized. | Line 186 |
|  | 12. Line 176: Clarify whether variables included in the logistic regression were significant in the univariate analysis. Is the p-value = 0.25 or <=0.25? | Variables included in the logistic regression had a p-value equal to 0.25 or less (p=<=0.25) in the univariate analysis.  The improved methods section has incorporated an expanded explanation of the inclusion criteria for variables into the model. | Line 203-211 |
|  | 13. The authors should say they obtained ethical approval/permissions as opposed to "sought ..." By just seeking it is not apparent whether they got it | The comment is well appreciated.  Informed written consent was obtained from all the participants.  The statement has been revised for better clarity. | Line 214-Line 216 |
|  | 14. Table 1, the number of cases and controls is not consistent for the variables. Instead of 94 cases, some are 93 and some are 91, and similarly, the total for controls is sometimes 90 instead of 91. There is a need to ensure that the data is cleaned before analysis. | The observation is appreciated    This was a case of missing data points for some variables, hence, missing data was treated as missing completely at random. |  |
|  | 16. Line 209-210, the sentences should read "...were less likely to contract malaria" to make sense. | This sentence has been revised to read :  “Those who slept in a room sprayed with IRS chemicals and those who wore clothes covering the whole body when conducting night outdoor activities were less likely to contract malaria.**”** | Lines 255-262 |
|  | 17. Table 2 includes the two-by-two table for the risk factors | Table 2 has been revised to incorporate a 2x2 table format | Line 273 |
|  | 18. It would be important for authors to discuss why traditionally protective factors, e.g., owning a treated net and sleeping under it, are not significant in this population. | We acknowledge that it is biologically plausible that sleeping under a net is protective against mosquito bites; however, this protective effect could be limited by some factors as shown in other studies cited in the text: damaged nets, frequent washing of treated nets, and expired net chemicals. In the study area, the nets in possession were not sourced from a malaria program. They were self-acquired. The integrity and efficacy of such nets is not guaranteed. ITN/ LLINS require replacement after certain period of time. The nets in this study could have exceeded their life span. Assessing the state of the nets and testing the chemical efficacy of the available LLINS was beyond the scope of the study, however, it is likely the cited factors that limit the protective of nets in other studies could also be responsible in the study area. | Line 361-371 |
|  | 19. In Table 3, the variable "engages in morning and night church activities" is not there in the univariate table or has been coined differently. | The text in Tables 2 and 3 has been harmonized.  The variable in both tables should read  Engages in night and morning outdoor religious activities. |  |
|  | 20. Table 4, on the question of the effectiveness of chemicals to spray homes, how did the authors expect the participants to know? Were you looking for their perceptions? | The text has been revised  The question was looking for perspectives of study participants on IRS chemical efficacy.  This question wanted to establish based on their viewpoint if they though the IRS chemicals were potent and offered protection | Line 308 |
|  | 21. The authors did not discuss the qualitative finding that this community resides in the fields far away from their sprayed homesteads as this is critical where the protective intervention is IRS? Is this a suitable intervention? | The point is well appreciated. The discussion has been expanded to incorporate the impact of this farming practice on the protective effect of IRS. | Line 395-399 |
|  | 22. The practice of wearing long clothes that cover the whole body should be discussed in light of the high temperatures in this area | The discussion on elevated temperatures has been made. | Line 372-382 |
|  | **REVIEWER # 3** |  |  |
|  | 1. The authors identified outdoor activities in the early night as a major driver for transmission in the Mudzi district (see results). This finding will be more significant if supported by secondary data on entomology, for example, dating on vector biting behaviour in the study site. | Additional details on the district's existing malaria vector feeding and resting behavior based on routine longitudinal entomological surveillance have been added  “The following vectors were identified in the district through longitudinal vector surveillance programs: *An. gambiae, An. funestus and An. arabiensis*  The established vectors have shown high preferences for human blood meal and indoor and outdoor feeding behavior.” | Line 398-400 |
|  | 2. The authors also identified Farming (gardening) activity as a driver for transmission. In the discussion, the authors associate these gardening activities with “man-made” mosquito breeding sites. As “farming activities” is an important human socio-economic activity that supports the life of the society, it is important to promote a vector-unfriendly farming system by avoiding the formation of water bodies that support larval development (larval source management | The point is well noted. As currently practiced in this district, garden farming relies on small bodies of water for watering purposes. Other community members utilize permanent communal wetlands after the rainy season. We have recommended larviciding as a sustainable intervention to treat small stagnant water bodies in and around garden fields as per the WHO vector control guidelines. | Line 405-407 |
|  |  |  |  |
