# Supplementary material for: Risk factors for contracting malaria in six wards of Mudzi District, Zimbabwe: A case control-study
Source: PLoS One. 2025 Aug 7;20(8):e0329093. doi: 10.1371/journal.pone.0329093 (PMC12331041; doi:10.1371/journal.pone.0329093)
Supplement: Attachment — Submitted filename: Dear Editor.docx [file pone.0329093.s006.docx]

Dear Editor

I have reviewed the manuscript and I have no further comments

Regards

Addmore Chadambuka
