# Supplementary material for: Risk factors for contracting malaria in six wards of Mudzi District, Zimbabwe: A case control-study
Source: PLoS One. 2025 Aug 7;20(8):e0329093. doi: 10.1371/journal.pone.0329093 (PMC12331041; doi:10.1371/journal.pone.0329093)
Supplement: Attachment — Submitted filename: Response_to_Reviewers_auresp_2.docx [file pone.0329093.s008.docx]

Response to Reviewers

| **Reviewer Comment** | **Response** | **Section** |
| --- | --- | --- |
| Line 82 - 83: The Anopheles gambiae complex is identified as the vector transmitting malaria. What behaviors of this vector have been observed at the study site in earlier research? Additionally, what is the current status of insecticide resistance, especially regarding those used in indoor residual spraying (IRS)? | Routine Insecticide Resistance, Wall cone Bioassay, and Vector Bionomics are routinely conducted in the study district and other malaria endemic districts in Zimbabwe.  Entomological surveillance reports have shown a predominant outdoor resting and outdoor biting preference for *An. gambiae ss, An. arabiensis ss and An. funestus ss*  Routine wall cone bioassay after Indoor residual spraying activities has shown chemical efficacy/decal rates consistent with WHO thresholds.  Resistance has not yet been identified for the pyrethroids and neonicotinoids, IRS chemicals in use.  Based on the national entomological surveys in the district, peak biting periods were established to be early evening, midnight, and early morning.    The vector behaviour, composition, and feeding and resting behaviour in the study district are well documented in the two (2) cited National Entomological Reports published in 2019 and 2022  The reports were originally available on the PMI Vector Link Websites; however, currently, the documents are not available online. Since the projects were USAID-funded, the current freeze could have affected their availability.  I have provided a link to a personal Google Drive link for the two (2) documents.  Google Drive Link: 2022 Report: <https://drive.google.com/file/d/1tQ1q8M3NRnYxzZJit5tzDWu8lv_XUGZt/view?usp=sharing>  2019 Report: <https://drive.google.com/file/d/1UzGIGvVULBTaxxq2ZZya707C6Su5i_PL/view?usp=sharing>  The background and discussion sections have been modified to capture the vital vector bionomics information from the entomological surveys.t | Line 84-Line 86 |
| Line 84 - 88: It is mentioned that Mudzi is one of the four districts in Zimbabwe where malaria transmission still occurs despite the implementation of an IRS program (with high performance) over the past five years. It is added that despite a significant decrease in cases, malaria remains the leading cause of morbidity in the district. However, no figures are provided on the extent of the decrease in cases, the number of remaining cases, or the proportion of the malaria burden among the total population (e.g., in the API). | This comment is well appreciated  Additional information on trends in incidence, API, and IRS coverages has been added to the text. Additional **Supplementary files 2 and 3** have been attached.  The revised text now reads:  *Malaria burden has significantly declined in Zimbabwe since 2000; however, high-burden districts like Mudzi, located on the country’s border regions, continue to record high malaria incidence rates and Annual Parasite Index (API), ranging between 100 and 200/1000 population between 2016 and 2021(32,38,39)* ***Supplementary File 3****. Entomology surveillance has shown the absence of insecticide resistance to IRS chemicals used in the national malaria vector control program (36,40). Efficacy testing results have also been consistently within the WHO pesticide monitoring thresholds (36,40).* | Line 80-Line 82  Line 95-98 |
| Lines 90 - 93: This study is part of a larger project to evaluate the entomological, social, and health system determinants of malaria transmission in Mudzi. To better understand how the transmission mechanisms will be explored through questionnaires and interviews, it is necessary to explain the relationship between this study and other parts of the project, particularly those related to entomology. | Additional text to link this study to the sub-studies has been added to the manuscript. The text now reads as follows:  *The primary factors for persistent malaria transmission in Mudzi, despite a high-performing IRS program, have not been fully explained. A broad project was conducted to evaluate the entomological, social, and health system primary factors responsible for persistent malaria transmission in Mudzi. Six sub-studies were conducted*   (i) *A study to identify vectoral composition and abundance in six wards.* (ii) *Focus Group Discussions to identify Knowledge and Attitude, and Perceptions among community members on malaria.* (iii) *Cross-sectional study incorporating Parasitaemia survey among artisanal miners and community members to identify practices, behaviour, and malaria Parasitaemia level* (iv) *Cross-border movement assessment among malaria cases* (v) *An assessment of the health system to establish health system capacities to deal with malaria.*   *The entomological studies were intended to identify the vector behaviors, whilst this nested case-control study was specifically conducted to establish the human social and behavioral determinants of malaria in six (6) wards of Mudzi District. Vector and human interaction patterns and mechanisms are critical determinants for malaria transmission.* | Line 95-107 |
| Line 279 - 285: Surprisingly, the questions regarding malaria knowledge did not include inquiries about local malaria vector mosquitoes, such as the abundance of mosquitoes inside and outside the house, the time and place of malaria transmission, and the habitat of Anopheles larvae in the vicinity of the house. | This valuable observation is highly appreciated.  Our questionnaire did not assess knowledge of local mosquito abundance, time and place of transmission, and the habitat of larvae, as those aspects were assessed using a different study tool.    The presence of breeding places around the households was assessed through direct observation using checklists.  Direct observation using a checklist was used to assess both the case and control environments and spaces around homes. The results on the nature and structure of living spaces and household environments were used to calculate measures of effect shown in **Table 2.** | Line 291  Table 2 |
| Line 300 - 302: The study indicates that many residents are traditional miners, which puts them at risk for malaria transmission in their work environment. Why wasn't this fact included in the malaria risk factor questionnaire? | This assessment of artisanal gold miners was provided for in the questionnaire. The results are in **Table 2 under Outdoor Activity.**  Those who engaged in artisanal mining were more likely to contract malaria compared to those who did not, with an = OR of 1.38. However, this association was not significant [95% CI, 0.36 -5.79], p>0.5. | Line 291 |
| Line 311 - 314: Many residents participate in religious meetings and services early in the morning and at night, often leaving them unprotected from mosquito bites. Are local malaria vectors also highly active in seeking blood meals during these times? | The comment is highly appreciated.  The aspects were studied elsewhere.  Google Drive Link 2022 National Entomological Report: <https://drive.google.com/file/d/1tQ1q8M3NRnYxzZJit5tzDWu8lv_XUGZt/view?usp=sharing>  2019 National Entomological Report: <https://drive.google.com/file/d/1UzGIGvVULBTaxxq2ZZya707C6Su5i_PL/view?usp=sharing>  Additional studies showing local vector behaviour preference for night outdoor biting have been cited in the manuscript.  Sande et al, <https://doi.org/10.1093/jme/tjw044>  Sande et al. : [https://journals.lww.com/jvbd/fulltext/2016/53020/Biting_behaviour_of_Anopheles_funestus_populations.5.aspx](C:\\Users\\FambiraiT\\Desktop\\UKZN\\PhD Papers and Theses\\Risk factors for contracting malaria in Mudzi\\RESUBMISSION PLOS ONE\\Manuscript_20052025.docx)  The outdoor activity coincides with peak biting phases of vectors obtaining in the district.  This is discussed in the text (discussion section):  *Long outdoor exposure coinciding with peak biting periods and the presence of efficient malaria vectors within the district increases the risk of contracting malaria. Routine vector surveillance data in the district have shown high abundance and distribution of An. gambiae sl, An. arabiensis and An. funestus with outdoor resting and feeding capabilities (36,39)*.  *Elevated night outdoor activity in a region with vectors with outdoor feeding and resting preferences elevates the risk of contracting malaria and suppresses the protective effect of IRS in the district* | Line 397-Line 401  Line 457-Line 459 |
| Line 315 - 317: Communities in wards 1 and 2 practice shifting cultivation and spend a significant amount of time during the rainy season (November-April) in agricultural areas, where malaria protection is very low. Malaria cases also increase during this period (lines 78 - 80). It should be specified with figures how many malaria cases increased/decreased according to local climatic conditions. | The comment is highly appreciated.  Additional information on seasonal trends about climatic changes has been added to the introduction section.  Supporting text reads:  *Weekly malaria incidence for malaria often increases from an average of 100 cases during the dry season to over 1500 cases per week during the wet season in the district* **Supplementary File** 1. | Line 80-91 |
| Lines 321 - 323: Cross-border migrants significantly contribute to malaria transmission, making it a critical issue to tackle. Do you have information on malaria cases linked to these migrants? If available, please include these figures in the manuscript. | The comment is highly appreciated.  This case-control study did not collect comprehensive data on cross-border movement; however, a separate study was conducted at selected health facilities to fully characterize cross-border movement among malaria cases.  For purposes of this study, the history of cross-border movement was assessed among cases and controls.  41/94 (43%) cases and 41/91(45%) controls reported a history of crossing the border into Mozambique. The association was not significant(OR 1.4 95% CI 0.37; 5.81) | Table 2 |
| Lines 328–330: If the district has implemented routine SBCC and Pre-IRS programs, why was the effectiveness of these programs not further explored in the malaria risk factor questionnaire? | The effectiveness of the routine SBCC and Pre-IRS was not assessed in this case-control questionnaire.  Based on our study findings, knowledge acquired through health workers, community health workers, and other channels was high. We evaluated the possible causes and implication of this mismatch in the discussion section.  Aspects of SBCC and community perception on IRS and its effectiveness were covered in a separate qualitative paper under review in a separate journal. The manuscript is available as a non-peer-reviewed pre-print (<https://www.medrxiv.org/content/10.1101/2025.04.30.25326649v2>). | Line 418- Line 425 |
